# Clinicopathological characteristics and outcomes of colorectal mucinous adenocarcinoma: a retrospective analysis from China

**DOI:** 10.3389/fonc.2024.1335678

**Published:** 2024-02-06

**Authors:** An Huang, Yong Yang, Zhuang Sun, Haopeng Hong, Jiajia Chen, Zhaoya Gao, Jin Gu

**Affiliations:** ^1^ Key Laboratory of Carcinogenesis and Translational Research (Ministry of Education/Beijing), Department of Gastrointestinal Surgery III, Peking University Cancer Hospital & Institute, Beijing, China; ^2^ Department of Gastrointestinal Surgery, Peking University Shougang Hospital, Beijing, China; ^3^ Department of General Surgery, Peking University First Hospital, Beijing, China

**Keywords:** colorectal cancer, mucinous adenocarcinoma, prognosis, nomogram, propensity score matching

## Abstract

**Background:**

Mucinous adenocarcinoma (MAC) is a unique subtype of colorectal cancer and its prognostic value remains controversial. This study aimed to compare the clinicopathological characteristics and prognostic differences between patients with MAC and non-mucinous adenocarcinoma (NMAC).

**Methods:**

674 patients with NMAC, 110 patients with adenocarcinoma with mucinous component (ACWM) and 77 patients with MAC between 2016-2019 were enrolled in the study. Univariate and multivariate Cox regression were performed to analyze the factors associated with prognosis. Predictive nomograms of overall survival (OS) and cancer-specific survival (CSS) for patients with colorectal adenocarcinoma were constructed. Confounding factors were eliminated by propensity score matching (PSM).

**Results:**

Compared with patients with NMAC, patients with MAC were more likely to have a tumor located at the proximal colon, present with a larger tumor diameter, more advanced T stage, higher frequency of metastasis, deficiency of mismatch repair, and elevated preoperative carcinoembryonic antigen. Patients with MAC were related to worse OS (HR=2.53, 95%CI 1.73-3.68, p<0.01) and CSS (HR=3.09, 95%CI 2.10-4.57, p<0.01), which persisted after PSM. Subgroup analysis demonstrated that patients with left-sided or stage III/IV MAC exhibited a comparatively worse OS and CSS than those with NMAC. Furthermore, in patients with stage II with a high-risk factor and stage III MAC, adjuvant chemotherapy was associated with an improved OS, CSS, and RFS.

**Conclusion:**

Compared with the NMAC phenotype, the MAC phenotype was an independent risk factor for poor prognosis in colorectal adenocarcinoma with worse OS and CSS, particularly patients with left-sided colorectal cancer and stage III/IV. However, patients with MAC can still benefit from adjuvant chemotherapy.

## 1 Introduction

The International Agency for Research on Cancer (IARC) estimated that China would have the highest numbers of new cancer cases (4.57 million) and cancer deaths (3 million) of any country in 2020, posing a huge burden on Chinese society (1). Data from the National Cancer Center of China revealed that in 2016, colorectal cancer (CRC) ranked second and fourth in incidence and mortality of all cancers in China, respectively, and the incidence was rising (2).

Mucinous adenocarcinoma (MAC), a distinctive subtype of colorectal adenocarcinoma (CRA), is defined as an adenocarcinoma in which more than 50% of the tumor tissue is composed of extracellular mucus according to pathological characteristics, and when the extracellular mucinous component accounts for less than 50% of the tumor tissue, it is called adenocarcinoma with mucinous component (ACWM) (3). More than 90% of CRC is CRA, and 1.6%–25.4% are reported as MAC (4). MAC has a clinicopathological profile distinct from non-mucinous adenocarcinoma (NMAC). It is more commonly seen in patients with early-onset CRC (5, 6), and often has proximal colon involvement (7, 8), larger diameter, and more advanced TNM stage at diagnosis (9). Additionally, MAC is distinguished by extracellular mucus rich in mucins such as MUC2 and MUC5AC (10), and exhibits a higher frequency of KRAS and BRAF mutations, deficiency of mismatch repair (dMMR), and CpG island methylator phenotype (11).

Unfortunately, current guidelines do not consider the mucus phenotype when guiding clinicians in making treatment decisions, and its prognostic value remains controversial. Several studies have identified MAC as a poor prognostic factor for patients with CRC (12, 13), while others have suggested no difference in survival between MAC and NMAC (14–16). Moreover, in left-sided colon and rectal cancer, 5-year overall survival (OS) is worse in patients with MAC than in patients with NMAC (43.8% vs. 78.2% [p=0.01] and 30.9% vs. 85.1% [p<0.01], respectively), while in right-sided colon cancer, there is no significant difference in 5-year OS between patients with MAC and NMAC (75.3% vs. 75.3%, p=0.42) (17). In this study, we retrospectively analyzed a large number of cases of CRA to compare clinicopathological features and prognostic differences among patients with MAC, ACWM, and NMAC.

## 2 Materials and methods

### 2.1 Patients

We collected clinicopathological information on patients who underwent CRC surgery at Peking University Shougang Hospital between January 2016 and December 2019. We defined NMAC as an adenocarcinoma in which extracellular mucus accounted for less than 5% of the tumor volume. Inclusion criteria included patients diagnosed with CRC between January 2016 and December 2019 at Peking University Shougang Hospital and the pathological diagnosis of the surgical specimen was adenocarcinoma. Exclusion criteria were: pathological types of CRC other than NMAC, ACWM (extracellular mucus accounting for more than 5% and less than 50% of tumor volume), and MAC (extracellular mucus accounting for 50% or more of tumor volume) (18); multiple primary cancers; history of malignancy; familial adenomatous polyposis. We registered on the official website of China Clinical Trial Registration Center (ChiCTR2300076785).

### 2.2 Clinicopathological characteristics and follow-up

Clinicopathological characteristics were obtained from patient medical records and pathology reports. Tumors in the cecum, ascending colon, and the right two-thirds of the transverse colon were considered right-sided CRC. Tumors in the left one-third of the transverse colon, descending colon, sigmoid colon, and rectum were considered left-sided CRC. TNM stage was determined using the American Joint Committee on Cancer (AJCC) Cancer Staging Manual (8th edition, 2017). Carcinoembryonic antigen (CEA) and carbohydrate antigen 19-9 (CA19-9) levels above the upper limit of normal were considered elevated. OS was defined as the time from surgery to death from any cause. Cancer-specific survival (CSS) was defined as the time from surgery to death from CRC. Recurrence-free survival (RFS) was defined as the time from surgery to first recurrence, metastasis, or death from any cause. Risk factors for patients with stage II CRC include poor histologic differentiation (grade III or IV) with proficiency of mismatch repair (pMMR) or microsatellite stability, T4, vascular or lymphatic vessel invasion (LVI), preoperative intestinal obstruction or perforation, insufficient lymph nodes (less than 12), perineural invasion (PNI), and elevated or undeterminable resection margin (19). Patients were followed up by telephone.

### 2.3 Construction of nomograms

We integrated survival time, survival status, and 13 factors (sex, age, tumor site, tumor diameter, pathological type, T stage, N stage, M stage, LVI, PNI, mismatch repair status, preoperative CEA and CA19-9) and used the rms package in R (Version 4.1.2; R Foundation for Statistical Computing, Vienna, Austria) based on the Cox method to create predictive nomograms of OS and CSS.

### 2.4 Propensity score matching

To diminish confounding factors and achieve a better comparison of the prognosis of patients with MAC and NMAC, we used the MatchIt package in R (Version 4.1.2) to conduct PSM on nine covariates (sex, age, tumor site, tumor diameter, TNM stage, LVI, PNI, mismatch repair status, and neoadjuvant chemoradiotherapy) based on the nearest neighbor method. The matching ratio of patients with MAC and NMAC was 1:3, and the caliper value was 0.1.

### 2.5 Statistical analysis

Statistical analysis was performed using IBM SPSS statistics Version 22.0 (IBM Corporation, Armonk, NY, USA), and Cox proportional risk regression model was used for univariate and multivariate analysis of OS and CSS. A Chi-square or Fisher’s exact test was applied to compare clinicopathological characteristics. OS, CSS, and RFS curves were estimated using the Kaplan–Meier method and the log-rank test was used for between-group comparisons. All reported p-values were two-tailed and significant at p<0.05.

## 3 Results

### 3.1 Clinicopathological characteristics

**Figure 1:**
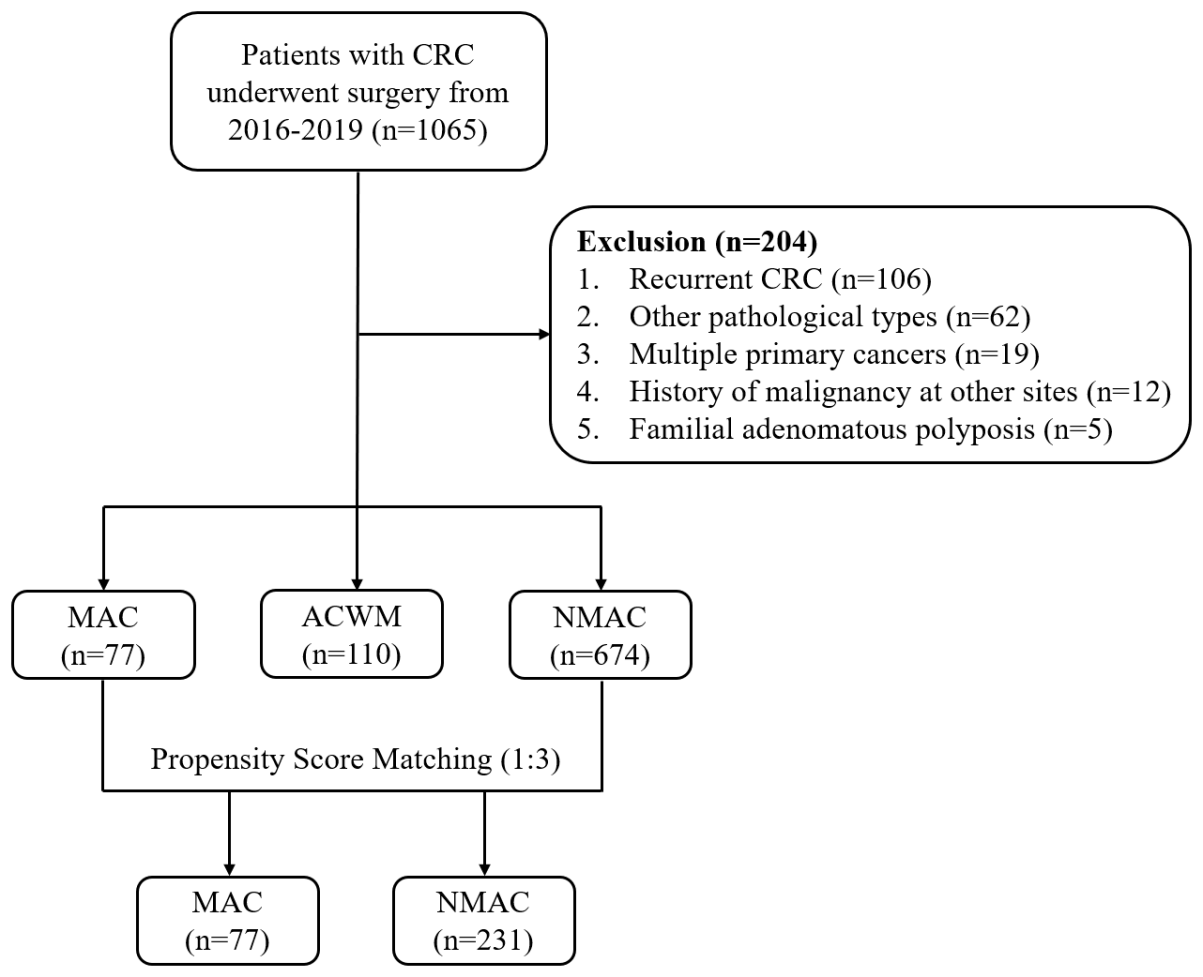
Study flow chart. CRC, colorectal cancer; MAC, mucinous adenocarcinoma; ACWM, adenocarcinoma with mucinous component; NMAC, non-mucinous adenocarcinoma.

Clinical data from 1065 patients were collected. Based on the screening criteria, 204 patients were excluded, including 106 patients with recurrence, 62 with other pathological types, 19 with multiple primary cancers, 12 with a history of malignancy at other sites, and five with familial adenomatous polyposis (**Figure 1**). Ultimately, 861 patients were included in the analysis. The median follow-up time was 44.13 months (interquartile range, 21.73–58.33).

Patients were divided into NMAC, ACWM, and MAC groups according to postoperative pathological results (674 [78.28%], 110 [12.78%], and 77 [8.94%] patients, respectively) (**Table 1**). Compared with patients with NMAC, patients with MAC were more likely to have a tumor located in the proximal colon, a larger tumor diameter (p<0.01), more advanced T stage (p<0.01), higher frequency of metastasis (p=0.02), dMMR (p<0.01), and elevated preoperative CEA (p<0.01). Although the incidence of liver metastasis did not differ significantly between patients with MAC and NMAC, peritoneal dissemination was more common in patients with MAC (18.18% vs. 2.08%, p<0.01). In patients with ACWM, tumors were more likely to be located in the proximal colon (p<0.01), be larger (p<0.01), and have a more advanced T stage (p<0.01), higher frequency of dMMR (p<0.01), and elevated preoperative CA19-9 (p=0.04) than patients with NMAC.

**Table 1:** Clinicopathological characteristics of patients with MAC, ACWM, and NMAC.

	MAC (n=77)	ACWM (n=110)	NMAC (n=674)	P
Age				0.31
≥50	61 (79.22%)	92 (83.64%)	577 (85.61%)	
<50	16 (20.28%)	18 (16.36%)	97 (14.39%)	
Sex				0.61
Male	48 (62.34%)	62 (56.36%)	412 (61.13%)	
Female	29 (37.66%)	48 (43.64%)	262 (38.87%)	
Tumor site				<0.01
Right-sided colon	26 (33.77%)	50 (45.45%)	114 (16.91%)	
Left-sided colon	16 (20.78%)	21 (19.09%)	168 (24.93%)	
Rectum	35 (45.45%)	39 (35.45%)	392 (58.16%)	
Tumor diameter (cm)				<0.01
≤5	30 (38.96%)	59 (53.64%)	488 (72.40%)	
>5	47 (61.04%)	51 (46.36%)	186 (27.60%)	
LVI				0.19
No	56 (72.73%)	90 (81.82%)	498 (73.89%)	
Yes	21 (27.27%)	20 (18.18%)	176 (26.11%)	
PNI				0.36
No	50 (64.94%)	79 (71.82%)	490 (72.70%)	
Yes	27 (35.06%)	31 (28.18%)	184 (27.30%)	
T stage				<0.01
T1, T2	4 (5.19%)	9 (8.18%)	147 (21.81%)	
T3, T4	73 (94.81%)	101 (91.82%)	527 (78.19%)	
N stage				0.18
N0	32 (41.56%)	54 (49.09%)	353 (52.37%)	
N1, N2	45 (58.44%)	56 (50.91%)	321 (47.63%)	
M stage				0.04
M0	57 (74.03%)	96 (87.27%)	570 (84.57%)	
M1	20 (25.97%)	14 (12.73%)	104 (15.43%)	
TNM stage				<0.01
I	2 (2.60%)	7 (6.36%)	120 (17.80%)	
II	27 (35.06%)	47 (42.73%)	213 (31.60%)	
III	28 (36.36%)	42 (38.18%)	237 (35.16%)	
IV	20 (25.97%)	14 (12.73%)	104 (15.43%)	
MMR status				<0.01
pMMR	62 (80.52%)	92 (83.64%)	634 (94.07%)	
dMMR	15 (19.48%)	18 (16.36%)	40 (5.93%)	
Peritoneal metastasis				<0.01
No	63 (81.82%)	107 (97.27%)	660 (97.92%)	
Yes	14 (18.18%)	3 (2.73%)	14 (2.08%)	
Synchronous liver metastasis				0.23
No	68 (88.31%)	102 (92.73%)	586 (86.94%)	
Yes	9 (11.69%)	8 (7.27%)	88 (13.06%)	
Neoadjuvant therapy				0.40
No	69 (89.61%)	102 (92.73%)	596 (88.43%)	
Yes	8 (10.39%)	8 (7.27%)	78 (11.57%)	
Preoperative CEA				0.03
Elevated	46 (59.74%)	52 (47.27%)	294 (43.62%)	
Normal	31 (40.26%)	58 (52.73%)	380 (56.38%)	
Preoperative CA19-9				0.07
Elevated	19 (24.68%)	29 (26.36%)	122 (18.10%)	
Normal	58 (75.32%)	81 (73.64%)	552 (81.90%)	
Postoperative chemotherapy				0.41
No	24 (31.17%)	41 (37.27%)	274 (40.65%)	
Yes	40 (51.95%)	50 (45.45%)	312 (46.29%)	
Unknown	13 (16.88%)	19 (17.27%)	88 (13.06%)	

MAC, mucinous adenocarcinoma; ACWM, adenocarcinoma with mucinous component; NMAC, non-mucinous adenocarcinoma; LVI, lymphatic vessel invasion; PNI, perineural invasion; pMMR, proficiency of mismatch repair; dMMR, deficiency of mismatch repair; CEA, carcinoembryonic antigen; CA19-9, carbohydrate antigen 19-9.

ACWM and MAC showed similar clinicopathological features for tumor site, T stage, and mismatch repair (MMR) status; however, patients with MAC had larger tumor diameter (p=0.048) and were more prone to metastasis (p=0.02), especially peritoneal metastasis (p<0.01).

### 3.2 Identification of prognostic factors and construction of nomograms

Univariate Cox regression analysis revealed that a tumor diameter above 5 cm, LVI, PNI, advanced TNM stage, MAC, elevated preoperative CEA, and elevated preoperative CA19-9 were risk factors for poor OS and CSS (**Table 2**). In addition, CSS was significantly better in patients with dMMR than in patients with pMMR (HR=0.61, 95%CI 0.32–1.17, p<0.01). When all the significant factors predicted in the univariate Cox regression model were taken into the multivariable survival analysis, a tumor diameter greater than 5 cm, PNI, advanced N and M stage, MAC, and elevated preoperative CEA and CA19-9 were risk factors for poor OS and a tumor diameter greater than 5 cm, PNI, advanced N and M stage, MAC, pMMR and elevated preoperative CA19-9 were risk factors for poor CSS. Furthermore, elevated preoperative CEA was also involved in poor OS (HR=1.51, 95%CI 1.09–2.10, p=0.01). Consequently, MAC was an independent prognostic factor for OS as well as CSS.

**Table 2:** Univariate and multivariate Cox regression analysis of OS and CSS in patients with colorectal adenocarcinoma.

Variables		OS						CSS					
		Univariate			Multivariate			Univariate			Multivariate		
		HR	95% CI	P	HR	95% CI	P	HR	95% CI	P	HR	95% CI	P
Age													
<50		REF						REF					
≥50		1.34	0.87–2.08	0.18				1.19	0.751–1.88	0.46			
Sex													
Male		REF						REF					
Female		1.00	0.75–1.34	0.99				1.09	0.79–1.49	0.60			
Tumor site													
Left–sided		REF						REF					
Right–sided		1.08	0.78–1.50	0.64				0.99	0.69–1.44	0.97			
Tumor diameter(cm)													
≤5		REF			REF			REF			REF		
>5		2.11	1.59–2.79	<0.01	1.75	1.30–2.37	<0.01	2.36	1.73–3.22	<0.01	2.13	1.53–2.97	<0.01
LVI													
No		REF			REF			REF			REF		
Yes		2.51	1.88–3.35	<0.01	1.07	0.76-1.52	0.69	2.93	2.14–4.02	<0.01	1.22	0.83-1.78	0.31
PNI													
No		REF			REF			REF			REF		
Yes		3.02	2.28–4.00	<0.01	1.51	1.09–2.09	0.01	3.49	2.56–4.76	<0.01	1.66	1.16–2.38	0.01
T stage													
T1		REF			REF			REF			REF		
T2		3.43	0.43–27.05	0.24	2.78	0.35–22.03	0.41	2.30	0.3–19.1	0.44	1.70	0.20-14.15	0.63
T3		10.62	1.48–75.96	0.02	4.14	0.39-36.62	0.16	8.73	1.2–62.6	0.03	2.92	0.40-21.40	0.29
T4		29.63	4.10–214.14	<0.01	6.73	0.91–49.94	0.06	24.82	3.42–179.98	<0.01	4.55	0.60-34.34	0.14
N stage													
N0		REF			REF			REF			REF		
N1		2.181	1.54–3.10	<0.01	1.46	1.00-2.12	0.05	2.52	1.70–3.72	<0.01	1.61	1.06-2.46	0.03
N2		4.47	3.16–6.32	<0.01	1.90	1.23-2.95	<0.01	4.95	3.35–7.32	<0.01	1.87	1.14-3.06	0.01
M stage													
M0		REF			REF			REF			REF		
M1		5.057	3.74–6.83	<0.01	2.51	1.78-3.53	<0.01	5.67	4.09–7.85	<0.01	2.43	1.66-3.55	<0.01
TNM stage													
I		REF						REF					
II		3.16	1.34–7.43	<0.01				3.38	1.19–9.60	0.02			
III		6.55	2.86–14.99	<0.01				8.17	2.98–22.39	<0.01			
IV		20.80	9.00–48.09	<0.01				27.51	9.97–75.92	<0.01			
Pathological type													
NMAC		REF			REF			REF			REF		
ACWM		0.95	0.61–1.47	0.80	0.82	0.53–1.29	0.40	0.82	0.49–1.38	0.46	0.72	0.43–1.23	0.24
MAC		2.53	1.74–3.68	<0.01	1.87	1.27–2.76	<0.01	3.09	2.09–4.55	<0.01	2.55	1.68–3.85	<0.01
MMR status													
pMMR		REF						REF			REF		
dMMR		0.65	0.37–1.14	0.13				0.61	0.32–1.17	<0.01	0.45	0.23–0.89	0.02
Preoperative CEA													
Normal		REF			REF			REF			REF		
Elevated		2.95	2.19–3.97	<0.01	1.57	1.13–2.18	0.01	2.71	1.96–3.75	<0.01	1.27	0.88-1.83	0.20
Preoperative CA19–9													
Normal		REF			REF			REF			REF		
Elevated		3.82	2.86–5.10	<0.01	1.70	1.21–2.38	<0.01	4.13	3.01–5.68	<0.01	1.97	1.35–2.86	<0.01

OS, overall survival; CSS, cancer-specific survival; MAC, mucinous adenocarcinoma; ACWM, adenocarcinoma with mucinous component; NMAC, non-mucinous adenocarcinoma; LVI, lymphatic vessel invasion; PNI, perineural invasion; pMMR, proficiency of mismatch repair; dMMR, deficiency of mismatch repair; CEA, carcinoembryonic antigen; CA19-9, carbohydrate antigen 19-9.

**Figure 2:**
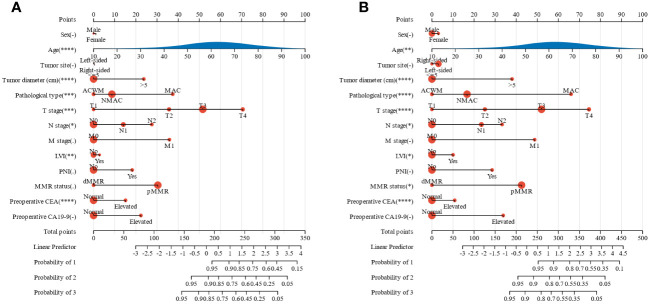
Nomograms for predicting the prognosis of patients with colorectal adenocarcinoma. **(A)** OS, **(B)** CSS. OS, overall survival; CSS, cancer-specific survival; MMR, mismatch repair; CEA, carcinoembryonic antigen; CA19-9, carbohydrate antigen 19-9; MAC, mucinous adenocarcinoma; ACWM, adenocarcinoma with mucinous component; NMAC, non-mucinous adenocarcinoma; LVI, lymphatic vessel invasion; PNI, perineural invasion. *, p<0.05; **, p<0.01; ***, p<0.001; ****, p<0.0001.

To effectively predict the survival of patients with colorectal adenocarcinoma, we constructed two nomograms based on 13 factors to predict OS and CSS (**Figures 2A, B**). The C-index of the nomogram for predicting OS and CSS was 0.80 and 0.82 (p<0.01), respectively. The calibration curves also suggested that our nomograms exhibited promising performance in predicting 1-year, 2-year, and 3-year OS, as well as CSS (**Supplementary Figure 1**).

### 3.3 Survival analysis

**Figure 3:**
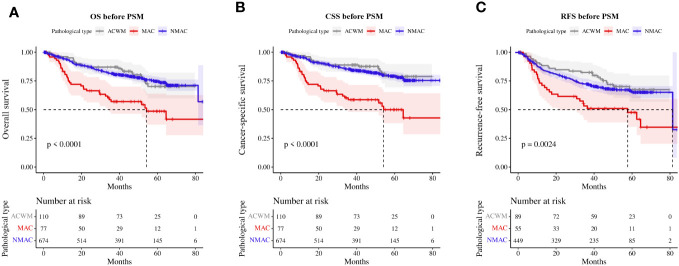
Survival rate of patients with MAC, ACWM, and NMAC. **(A)** OS, **(B)** CSS, **(C)** RFS in stage II and III patients. MAC, mucinous adenocarcinoma; ACWM, adenocarcinoma with mucinous component; NMAC, non-mucinous adenocarcinoma; OS, overall survival; CSS, cancer-specific survival; RFS, recurrence-free survival.

Univariate and multivariate Cox regression analyses indicated that MAC was an independent prognostic factor for OS and CSS in patients with CRA. A Kaplan–Meier survival analysis showed that OS (HR=2.53, 95%CI 1.73–3.68, p<0.01, **Figure 3A**) and CSS (HR=3.09, 95%CI 2.10–4.57, p<0.01, **Figure 3B**) were worse in MAC than NMAC, while OS (HR=0.95, 95%CI 0.61–1.47, p=0.81) and CSS (HR=0.82, 95%CI 0.48–1.38, p=0.45) were not significantly different between patients with ACWM and NMAC. The 1-year, 2-year, and 3-year OS rates for patients with MAC versus NMAC were 79.10% vs. 93.63% (HR=3.44, 95%CI 1.90–6.24, p<0.01), 66.38% vs. 87.14% (HR=2.99, 95%CI 1.89–4.72, p<0.01), and 57.02% vs. 81.44% (HR=2.75, 95%CI 1.83–4.12, p<0.01), respectively (**Supplementary Table 1**). Regarding CSS, the 1-year, 2-year, and 3-year CSS rates for patients with MAC versus NMAC were 79.10% vs. 95.23% (HR=4.61, 95%CI 2.47–8.60, p<0.01), 66.38% vs. 89.70% (HR=3.77, 95%CI 2.35–6.05, p<0.01), and 58.60% vs. 85.22% (HR=3.40, 95%CI 2.23–5.18, p<0.01), respectively.

Furthermore, we analyzed RFS in patients with stage II and III CRA. Compared with patients with NMAC, patients with MAC presented with worse RFS (HR=2.10, 95%CI 1.48–2.99, p<0.01, **Figure 3C**), while there was no significant difference in RFS (HR=0.90, 95%CI 0.62–1.32, p=0.60) between patients with ACWM and NMAC.

### 3.4 Survival analysis after PSM

**Figure 4:**
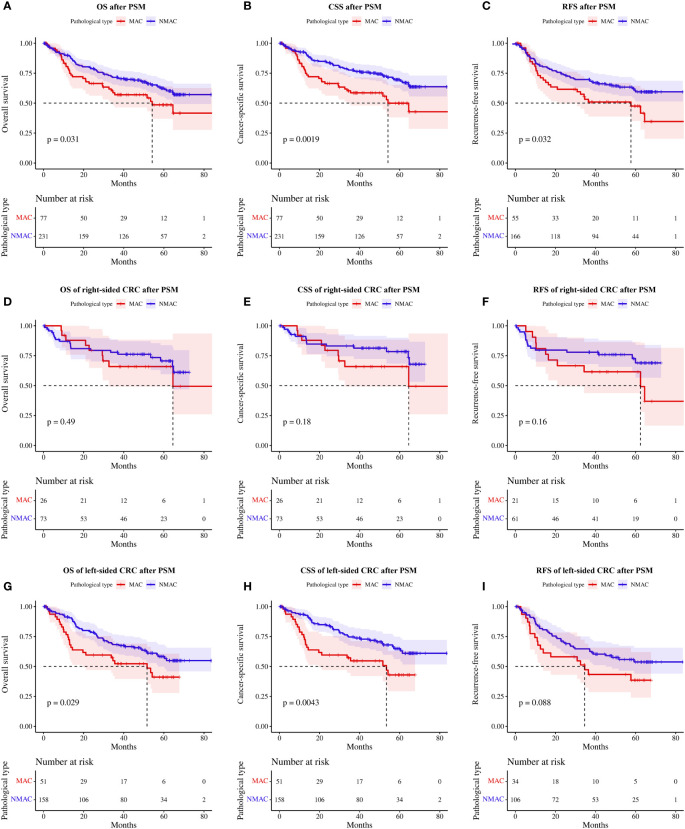
Survival rates of patients with MAC and NMAC after PSM **(A-C)**. **(A)** OS, **(B)** CSS, and **(C)** RFS in stage II and III patients. Survival rates of patients with right-sided MAC and NMAC after PSM **(D-F)**. **(D)** OS, **(E)** CSS, and **(F)** RFS in stage II and III patients. Survival rates of patients with left-sided MAC and NMAC after PSM **(G-I)**. **(G)** OS, **(H)** CSS, **(I)** RFS in stage II and III patients. MAC, mucinous adenocarcinoma; NMAC, non-mucinous adenocarcinoma; PSM, propensity score matching; OS, overall survival; CSS, cancer-specific survival; RFS, recurrence-free survival.

Given the significant differences in the clinicopathological characteristics of patients with MAC and NMAC, straightforward comparison of survival between the two groups would be biased; hence, we matched the two groups based on sex, age, tumor site, tumor diameter, TNM stage, LVI, PNI, mismatch repair status, and the presence of neoadjuvant chemoradiotherapy, with a matching ratio of 1:3. Seventy-seven patients with MAC and 231 patients with NMAC were eventually enrolled, with no significant differences in clinicopathological characteristics between the two groups after PSM (**Table 3**). One-year, 2-year, and 3-year OS rates for MAC versus NMAC after PSM were 79.10% vs. 89.92%, 66.38% vs. 78.80%, and 57.02% vs. 71.53%, respectively. RFS rates at 1, 2, and 3 years for stage II and III patients with MAC and NMAC after PSM were 81.58% vs. 73.10%, 73.75% vs. 61.54%, and 69.74% vs. 53.17%, respectively. Kaplan–Meier survival analysis of matched patients consistently indicated that OS (HR=1.56, 95%CI 1.04–2.35, p=0.03, **Figure 4A**) and CSS (HR=1.95, 95%CI 1.27–3.00, p<0.01, **Figure 4B**) were worse in patients with MAC. Meanwhile, RFS (HR=1.63, 95%CI 1.04–2.57, p<0.01, **Figure 4C**) was also worse in patients with stage II and III MAC than in those with NMAC.

**Table 3:** Clinicopathological characteristics of patients with MAC and NMAC after PSM.

		MAC (n=77)	NMAC (n=231)	P
Age				0.87
≥50		61 (79.22%)	185 (80.09%)	
<50		16 (20.78%)	46 (19.91%)	
Sex				0.89
Male		48 (62.34%)	142 (61.47%)	
Female		29 (37.66%)	89 (38.53%)	
Tumor site				0.73
Right-sided		26 (33.77%)	73 (31.60%)	
Left-sided		51 (66.23%)	158 (68.40%)	
Tumor diameter (cm)				0.55
≤5		30 (38.96%)	99 (42.86%)	
>5		47 (61.04%)	132 (57.14%)	
LVI				0.83
No		56 (72.73%)	165 (71.43%)	
Yes		21 (27.27%)	66 (28.57%)	
PNI				0.73
No		50 (64.94%)	145 (62.77%)	
Yes		27 (35.06%)	86 (37.23%)	
T stage				1.00
T1, T2		4 (5.19%)	12 (5.19%)	
T3, T4		73 (94.81%)	219 (94.81%)	
N stage				0.79
N0		32 (41.56%)	100 (43.29%)	
N1, N2		45 (58.44%)	131 (56.71%)	
M stage				0.76
M0		57 (74.03%)	175 (75.76%)	
M1		20 (25.97%)	56 (24.24%)	
TNM stage				0.98
I		2 (2.60%)	8 (3.46%)	
II		27 (35.06%)	81 (35.06%)	
III		28 (36.36%)	86 (37.23%)	
IV		20 (25.97%)	56 (24.24%)	
MMR status				0.37
pMMR		62 (80.52%)	196 (84.85%)	
dMMR		15 (19.48%)	35 (15.15%)	
Neoadjuvant therapy				1.00
No		69 (89.61%)	207 (89.61%)	
Yes		8 (10.39%)	24 (10.39%)	
Preoperative CEA				0.35
Elevated		46 (59.74%)	124 (53.68%)	
Normal		31 (40.26%)	107 (46.32%)	
Preoperative CA19-9				0.88
Elevated		19 (24.68%)	59 (25.54%)	
Normal		58 (75.32%)	172 (74.46%)	
Postoperative chemotherapy				0.89
No		24 (71.17%)	76 (32.90%)	
Yes		40 (51.95%)	121 (52.38%)	
Unknown		13 (16.88%)	34 (14.72%)	

MAC, mucinous adenocarcinoma; NMAC, non-mucinous adenocarcinoma; PSM, propensity score matching; LVI, lymphatic vessel invasion; PNI, perineural invasion; pMMR, proficiency of mismatch repair; dMMR, deficiency of mismatch repair; CEA, carcinoembryonic antigen; CA19-9, carbohydrate antigen 19-9.

Subgroup analysis of matched patients according to tumor site showed no significant differences in OS (HR=1.32, 95%CI 0.60–2.91, p=0.49, **Figure 4D**), CSS (HR=1.75, 95%CI 0.76–4.01, p=0.18, **Figure 4E**), and stage II/III patients’ RFS (HR=1.75, 95%CI 0.79–3.87, p=0.16, **Figure 4F**) between patients with MAC and NMAC in right-sided CRC. In contrast, in left-sided CRC, OS (HR=1.69, 95%CI 1.05–2.73, p=0.03, **Figure 4G**) and CSS (HR=2.05, 95%CI 1.24–3.39, p<0.01, **Figure 4H**) were significantly poorer in patients with MAC than in patients with NMAC. In patients with stage II/III left-sided CRC, RFS tended to be worse in patients with MAC, but the difference was not statistically significant (HR=1.61, 95%CI 0.93–2.80, p=0.09, **Figure 4I**).

**Figure 5:**
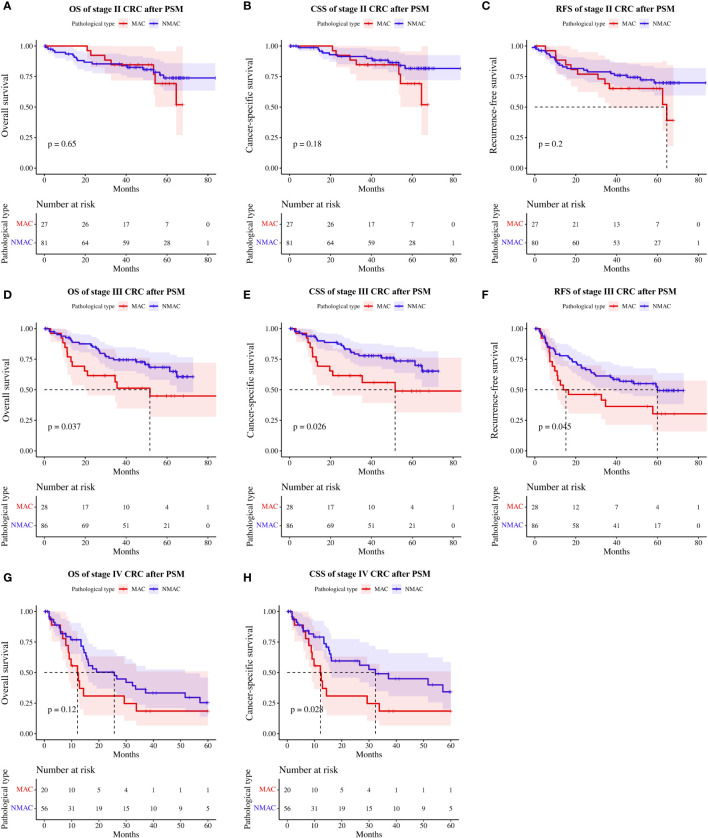
Survival rates of patients with MAC versus NMAC at different TNM stages after PSM. **(A)** OS in stage II, **(B)** CSS in stage II, **(C)** RFS in stage II, **(D)** OS in stage III, **(E)** CSS in stage III, **(F)** RFS in stage III, **(G)** OS in stage IV, and **(H)** CSS in stage IV. MAC, mucinous adenocarcinoma; NMAC, non-mucinous adenocarcinoma; OS, overall survival; CSS, cancer-specific survival; RFS, recurrence-free survival.

Subgroup analysis based on TNM staging revealed that neither OS (HR=1.23, 95%CI 0.51–2.97, p=0.65, **Figure 5A**), CSS (HR=1.91, 95%CI 0.74–4.94, p=0.18, **Figure 5B**), or RFS (HR=1.61, 95%CI 0.77–3.34, p=0.20, **Figure 5C**) were significantly different between patients with stage II MAC and NMAC. However, patients with stage III MAC had poorer OS (HR=2.02, 95%CI 1.03–3.95, p=0.04, **Figure 5D**), CSS (HR=2.20, 95%CI 1.08–4.47, p=0.03, **Figure 5E**), and RFS (HR=1.79, 95%CI 1.00–3.18, p=0.045, **Figure 5F**) than matched patients with NMAC. There was no significant difference in OS in stage IV MAC and stage IV NMAC patients (HR=1.67, 95%CI 0.87–3.19, p=0.12, **Figure 5G**), while CSS (HR=2.10, 95%CI 1.07–4.14, p=0.03, **Figure 5H**) was worse in stage IV MAC.

**Figure 6:**
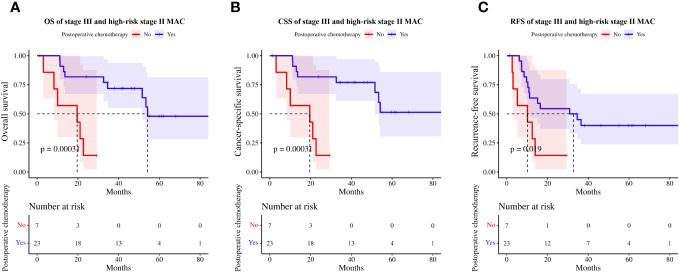
Effect of postoperative chemotherapy on survival in high-risk stage II and stage III patients with MAC. **(A)** OS, **(B)** CSS, **(C)** RFS. MAC, mucinous adenocarcinoma; OS, overall survival; CSS, cancer-specific survival; RFS, recurrence-free survival.

Regarding the role of adjuvant chemotherapy, patients with stage III and high-risk stage II MAC (**Supplementary Table 2**) who received adjuvant chemotherapy had prolonged OS (HR=0.13, 95%CI 0.04–0.48, p<0.01, **Figure 6A**), CSS (HR=0.13, 95%CI 0.04–0.48, p<0.01, **Figure 6B**), and RFS (HR=0.31, 95%CI 0.11–0.87, p=0.02, **Figure 6C**) compared to those who did not.

## 4 Discussion

MAC is a distinct subtype of CRC, and its prognostic value remains controversial. In this research, we collected clinicopathological information from 861 patients with CRA and divided them into three groups based on the amount of extracellular mucus. We discovered that patients with MAC tended to have tumors predominantly located in the right-sided colon with a larger diameter than patients with NMAC. Additionally, they tended to have an advanced T stage and a greater susceptibility to metastasis, particularly peritoneal metastasis, as well as a higher frequency of dMMR and elevated preoperative CEA.

Studies have suggested that MAC is more common in young female patients (16, 20). However, our findings demonstrated no significant differences in age or sex between patients with MAC and those with NMAC, which supports previous research (21, 22). Patients with MAC demonstrated similar characteristics to those with NMAC in terms of LVI, PNI, regional lymph node metastasis, and liver metastasis. However, they were more susceptible to metastasis, specifically peritoneal metastasis (23), indicating that metastatic processes may differ in MAC and NMAC. Peritoneal metastasis is a stepwise process that starts with the shedding of tumor cells from the surface of the primary tumor in most patients (24). Shedding of tumor cells is associated with cell adhesion molecules, such as E-cadherin, which undergo epithelial-mesenchymal transition and become more aggressive (25). In most solid tumors, increased interstitial fluid pressure further promotes spontaneous detachment of tumor cells (26). More than 50% mucin-rich mucus in MAC could be an important source of spontaneous detachment of tumor cells and peritoneal metastasis.

Tumor diameter, PNI, TNM stage, MAC phenotype, preoperative CEA, and CA19-9 were independent prognostic factors for OS, while tumor diameter, PNI, TNM stage, MAC phenotype, mismatch repair status, and preoperative CA19-9 were independent prognostic factors for CSS in patients with primary CRA. Therefore, we constructed two nomograms based on 13 factors, including MAC phenotype, to predict OS and CSS in patients with CRA.

Consistent with Foda et al. and Lin et al. (27, 28), our findings illustrated that prognosis of patients with ACWM was significantly better than that of patients with MAC but was comparable to that of patients with NMAC. ACWM presented an intermediate status between MAC and NMAC in terms of clinicopathological features. Most clinicopathological features of patients with ACWM, such as TNM stage and mismatch repair status, were similar to those of MAC, but the metastatic risk of ACWM was similar to that of NMAC, which may explain why patients with ACWM had a better prognosis than patients with MAC.

Both OS and CSS were worse in patients with MAC than in patients with NMAC. Certain researchers have proposed that the dissimilarity in prognosis can be attributed to the fact that patients with MAC are diagnosed at more advanced stages (29, 30). To ameliorate these factors, which may potentially cause variations in prognosis, we matched patients according to a 1:3 propensity score. After PSM, MAC patients continued to exhibit inferior results in OS and CSS compared to NMAC patients. Additionally, stage II and III MAC patients exhibited a decreased RFS compared to their NMAC counterparts. To further elucidate the source of the prognostic difference, we performed subgroup analysis according to tumor location and TNM stage. This revealed no discernible difference in prognosis between patients with right-sided MAC and NMAC. Conversely, patients with left-sided MAC exhibited a comparatively worse OS and CSS than those with NMAC, but there were no significant differences in RFS. Lan et al. (17) proposed that the dissimilarity could be attributed to the more advanced TNM stage among patients with left-sided MAC. Furthermore, our analysis revealed no notable distinctions in OS, CSS, and RFS between stage II MAC and NMAC. However, patients with stage III MAC had poorer OS, CSS, and RFS compared to those with NMAC, which is congruent with the findings of Kim et al. (31). Among stage IV patients, MAC patients had comparable OS to NMAC patients, but their CSS was inferior. We performed a search for studies comparing survival in patients with MAC and NMAC from 2018-2023 and enrolled 24 studies (7, 17, 22, 32–52), 17 of which compared OS and 11 of which compared CSS/DSS (**Supplementary Table 3**). 64.7% of studies suggested that patients with MAC had poorer OS than patients with NMAC, and 72.7% concluded that patients with MAC had worse CSS/DSS than patients with NMAC, with two of the four studies showing that this survival difference existed only in left-sided CRC or rectal cancer.

The current study confirmed that adjuvant chemotherapy for patients with stage II with a high-risk factor and stage III MAC was associated with improved OS, CSS, and RFS. Therefore, patients with MAC should be treated with adjuvant chemotherapy until better treatments are available. Nevertheless, these patients received chemotherapy with 5-fluorouracil in combination with oxaliplatin, and the effect of other chemotherapy regimens on patients with MAC requires further validation. Moreover, several studies have proposed MAC as one of the high-risk factors for patients with stage II CRC (53, 54), and regular postoperative chemotherapy should be considered for patients with stage II MAC, regardless of other high-risk factors. However, although most studies have revealed that patients with MAC could benefit from adjuvant chemotherapy (37, 55), the extent of the benefit appears to be limited (56, 57), which has always plagued clinicians. Potential mechanisms of chemotherapy resistance in MAC include physical barrier formed by mucus, inhibition of apoptosis, genetic alterations related to chemotherapeutic drug metabolism, enhancement of tumor cell stemness, and promotion of the epithelial-mesenchymal transition process (58). The reticular structure formed by the secreted mucus, as well as the extraordinary size of heavily O-glycosylated membrane-bound mucins such as MUC1 or MUC4, are capable of limiting drug intracellular entrance and immune recognition of tumor cell epitopes in antibody-based therapies (59). Resistance genes associated with 5-fluorouracil, oxaliplatin, and irinotecan, such as TYMP, ATP7B, SRPK1, ABCB1, and ABCG2, exhibit a higher frequency of somatic mutations (60), which may be partially responsible for chemotherapy resistance in patients with MAC. To overcome the challenges of poor prognosis and chemotherapy resistance in patients with MAC, future research should be devoted to the development of novel targeted drugs that address the molecular characteristics of MAC.

There are several limitations associated with the present study. We must be aware that this study is a retrospective single center study and although we used PSM to adjust for known confounding factors, some degree of selection bias cannot be excluded. Secondly, since the chemotherapy regimens recommended by the current guidelines are not differentiated according to mucus differentiation, there was no difference in chemotherapy regimens between our MAC and NMAC patients, and since we only analyzed the effect of chemotherapy on the prognosis of patients with stage II-III MAC, more studies are needed in the future to elucidate the effect of different chemotherapy regimens on patients with MAC. Thirdly, given that we are a retrospective study, 84.0% of our patients were stage I-III, and current guidelines only recommend genetic testing for stage IV patients, the vast majority of our patients had no information on genetic mutations. Some genetic mutations such as KRAS and BRAF also affect the prognosis of CRC patients, therefore, larger sample sizes and multi-center data are needed in the future to prove our findings.

In conclusion, Patients with MAC had significantly different clinicopathological characteristics and worse OS and CSS than patients with NMAC. The prognostic differences persisted after PSM, mainly in patients with left-sided CRC and stage III/IV. Patients with MAC can benefit from adjuvant chemotherapy; however, larger studies are needed to confirm these findings.

## Data availability statement

The original contributions presented in the study are included in the article/**Supplementary Material**. Further inquiries can be directed to the corresponding author.

## Ethics statement

The studies involving humans were approved by Peking University Shougang Hospital Medical Ethics Committee. The studies were conducted in accordance with the local legislation and institutional requirements. The ethics committee/institutional review board waived the requirement of written informed consent for participation from the participants or the participants’ legal guardians/next of kin because this is a retrospective study and patients will not be identified.

## Author contributions

AH: Data curation, Formal analysis, Writing – original draft. YY: Methodology, Writing – review & editing. ZS: Validation, Writing – review & editing. HH: Software, Writing – review & editing. JC: Writing – review & editing. ZG: Writing – review & editing. JG: Conceptualization, Funding acquisition, Supervision, Writing – review & editing.
