# Supplementary material for: Clinicopathological characteristics and outcomes of colorectal mucinous adenocarcinoma: a retrospective analysis from China
Source: Front Oncol. 2024 Feb 6;14:1335678. doi: 10.3389/fonc.2024.1335678 (PMC10878404; doi:10.3389/fonc.2024.1335678)
Supplement: Supplementary file 1 [file DataSheet_1.docx]

Supplementary Material

# Supplementary Figures and Tables

## Supplementary Figures


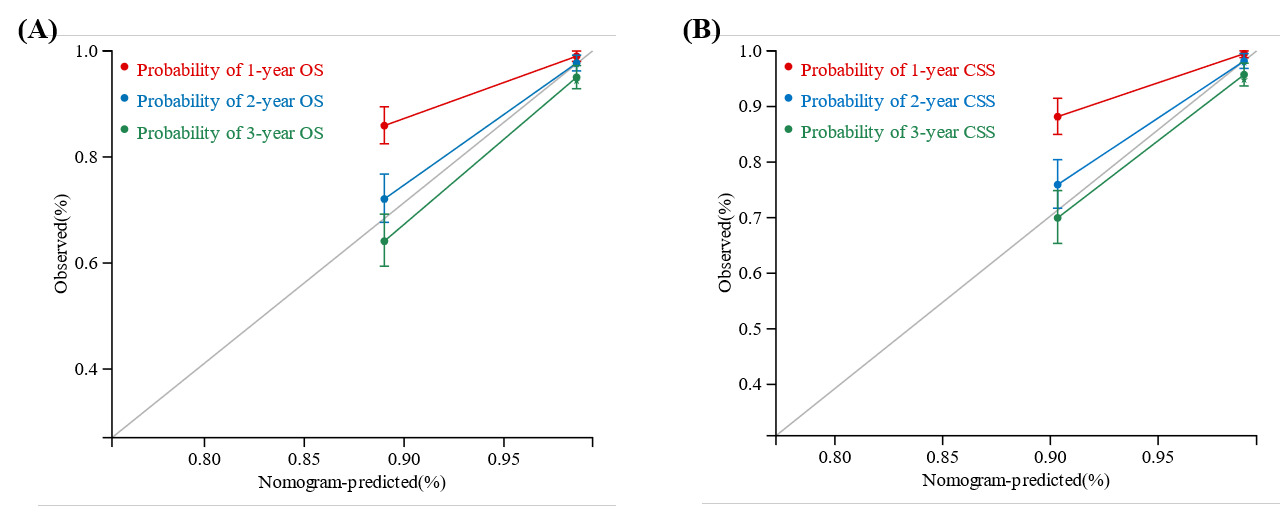


**Supplementary Figure 1.** Calibration curves for the nomogram. (A) OS, (B) CSS. OS, overall survival; CSS, cancer-specific survival.

## Supplementary Tables

**Supplementary Table 1.** Comparison of 1-year, 2-year, and 3-year OS and CSS in patients with MAC, ACWM, and NMAC.

|  | OS | | | |  | CSS | | | |
| --- | --- | --- | --- | --- | --- | --- | --- | --- | --- |
|  | HR | 95%CI | | P |  | HR | 95%CI | | P |
| 1-year |  |  |  |  |  |  |  |  |  |
| MAC vs. NMAC | 3.44 | 1.90–6.24 | | <0.01 |  | 4.61 | 2.47–8.60 | | <0.01 |
| ACWM vs. NMAC | 0.61 | 0.22–1.71 | | 0.34 |  | 0.62 | 0.19–2.03 | | 0.42 |
| MAC vs. ACWM | 5.66 | 1.88–17.07 | | <0.01 |  | 7.57 | 2.19–26.14 | | <0.01 |
| 2-year |  |  |  |  |  |  |  |  |  |
| MAC vs. NMAC | 2.99 | 1.89–4.72 | | <0.01 |  | 3.77 | 2.35–6.05 | | <0.01 |
| ACWM vs. NMAC | 0.99 | 0.55–1.77 | | 0.96 |  | 1.05 | 0.55–2.00 | | 0.88 |
| MAC vs. ACWM | 3.03 | 1.54–5.96 | | <0.01 |  | 3.59 | 1.76–7.33 | | <0.01 |
| 3-year |  |  |  |  |  |  |  |  |  |
| MAC vs. NMAC | 2.75 | 1.83–4.12 | | <0.01 |  | 3.40 | 2.23–5.18 | | <0.01 |
| ACWM vs. NMAC | 0.69 | 0.39–1.22 | | 0.20 |  | 0.74 | 0.40–1.39 | | 0.35 |
| MAC vs. ACWM | 3.93 | 2.05–7.55 | | <0.01 |  | 4.47 | 2.23–8.96 | | <0.01 |

OS, overall survival; CSS, cancer-specific survival; MAC, mucinous adenocarcinoma; ACWM, adenocarcinoma with mucinous component; NMAC, non-mucinous adenocarcinoma.

**Supplementary Table 2.** Clinicopathological characteristics of high-risk stage II and stage III patients with MAC.

|  | | High-risk stage II and stage III patients with MAC (n=38) |
| --- | --- | --- |
| Age | |  |
| ≥50 | | 31(81.58%) |
| <50 | | 7 (18.42%) |
| Sex | |  |
| Male | | 26 (68.42%) |
| Female | | 12 (31.58%) |
| Tumor site | |  |
| Right-sided | | 10 (26.32%) |
| Left-sided | | 28 (73.68%) |
| Tumor diameter (cm) |  | |
| ≤5 | | 13 (34.21%) |
| >5 | | 25 (65.79%) |
| LVI | |  |
| No | | 25 (65.79%) |
| Yes | | 13 (34.21%) |
| PNI | |  |
| No | | 19 (50.00%) |
| Yes | | 19 (50.00%) |
| TNM stage | |  |
| II | | 10 (26.32%) |
| III | | 28 (73.68%) |
| MMR status | |  |
| pMMR | | 30 (78.95%) |
| dMMR | | 8 (21.05%) |
| Neoadjuvant therapy | | |
| No | | 34 (89.47%) |
| Yes | | 4 (10.53%) |
| Preoperative CEA | | |
| Elevated | | 24 (63.16%) |
| Normal | | 14 (36.84%) |
| Preoperative CA19-9 | | |
| Elevated | | 9 (23.68%) |
| Normal | | 29 (76.32%) |
| Postoperative chemotherapy | | |
| No | | 7 (18.42%) |
| Yes | | 23 (60.53%) |
| Unknown | | 8 (21.05%) |
| OS  Alive  Dead  CSS  Alive  Dead  RFS  Without recurrence  With recurrence | | 21 (55.26%)  17 (44.74%)  22 (57.89%)  16 (42.11%)  16 (42.11%)  22 (57.89%) |

MAC, mucinous adenocarcinoma; LVI, lymphatic vessel invasion; PNI, perineural invasion; pMMR, proficiency of mismatch repair; dMMR, deficiency of mismatch repair; CEA, carcinoembryonic antigen; CA19-9, carbohydrate antigen 19-9; OS, overall survival; CSS, cancer-specific survival; RFS, recurrence-free survival.

**Supplementary Table 3.** Studies comparing the survival of patients with MAC and NMAC from 2018 to 2023.

| First author | Year | Country (data source) | N (MAC) | N (NMAC) | Tumor site | TNM stage | OS | | |  | DSS/CSS | | |
| --- | --- | --- | --- | --- | --- | --- | --- | --- | --- | --- | --- | --- | --- |
|  |  |  |  |  |  |  | HR | 95%CI | P |  | HR | 95%CI | P |
| Wang(32) | 2020 | U.S. | 2131 | 24039 | Colon | Stage IV | 1.05 | 1.00‐1.11 | 0.05 |  | 1.06 | 1.00‐1.12 | 0.49 |
| Zhu(33) | 2021 | U.S. | 40111 | 270070 | Right–sided colon | Stage I–IV |  |  |  |  | 0.96 | 0.04–0.99 | <0.01 |
|  |  |  | 15775 | 233964 | Left–sided colon | Stage I–IV |  |  |  |  | 1.16 | 1.12–1.21 | <0.01 |
|  |  |  | 12783 | 213756 | Rectum | Stage I–IV |  |  |  |  | 1.22 | 1.17–1.28 | <0.01 |
| Benesch(34) | 2020 | U.S. | 40232 | 243687 | Colon | Stage I–IV |  |  |  |  | 1.06 | 1.04–1.08 | <0.05 |
|  |  |  | 8845 | 110428 | Rectum | Stage I–IV |  |  |  |  | 1.35 | 1.31–1.39 | <0.05 |
| Li(35) | 2019 | U.S. | 9982 | 123501 | Colon and rectum | Stage I–II |  |  |  |  | 1.14 | 1.07–1.20 | <0.01 |
|  |  |  | 6922 | 64716 | Colon and rectum | Stage III |  |  |  |  | 1.22 | 1.16–1.27 | <0.01 |
|  |  |  | 3180 | 28317 | Colon and rectum | Stage IV |  |  |  |  | 1.13 | 1.08–1.18 | <0.01 |
| Dai(36) | 2019 | U.S. | 13035 | 61958 | Colon and rectum | Stage I–IV | 1.07 | 1.05–1.10 | <0.01 |  | 1.07 | 1.03–1.11 | <0.01 |
| Fields(37) | 2019 | U.S. | 35267 | 262759 | Colon | Stage II–III | 1.05 | 1.02–1.08 | <0.01 |  |  |  |  |
| Patel(38) | 2023 | U.S. | 86 | 424 | Right–sided colon | Stage I–IV |  |  |  |  | 0.89 | 0.63–1.27 | 0.46 |
|  |  |  | 50 | 344 | Left–sided colon | Stage I–IV |  |  |  |  | 1.12 | 0.79–1.82 | 0.39 |
|  |  |  | 37 | 246 | Rectum | Stage I–IV |  |  |  |  | 1.85 | 1.15–2.97 | 0.01 |
| Sheng(39) | 2019 | U.S. | 5689 | 15812 | Colon and rectum | Stage I–IV |  |  |  |  | 1.14 | 0.93–1.39 | 0.20 |
| Yu(40) | 2020 | U.S. | 6592 | 62384 | Colon | Stage III |  |  |  |  | 1.06 | 1.01–1.11 | 0.03 |
| Khan(41) | 2018 | U.S. | 277 | 1548 | Colon and rectum | Stage IV | 1.38 | 1.17–1.63 | <0.01 |  |  |  |  |
| Powers(42) | 2021 | U.S. | 12297 | 95096 | Colon | Stage III | 1.15 | 1.12–1.19 | <0.01 |  |  |  |  |
| Wu(43) | 2020 | U.S. | 190 | 3058 | Rectum | Stage II |  |  |  |  | 1.36 | 1.01–1.83 | 0.04 |
| Zhou(44) | 2023 | U.S. | 3079 | 25686 | Colon | Stage III | 1.12 | 1.03–1.23 | 0.01 |  |  |  |  |
| Bagante(45) | 2018 | U.S. | 16432 | 16432 | Colon and rectum | Stage I–IV | 1.08 | 1.05–1.12 | <0.01 |  |  |  |  |
| Wang(46) | 2019 | U.S. | 7003 | 42395 | Right–sided colon | Stage II–IV | 1.04 | 0.97–1.11 | 0.30 |  |  |  |  |
|  |  |  | 2262 | 29682 | Left–sided colon and rectum | Stage II–IV | 1.23 | 1.11–1.38 | <0.01 |  |  |  |  |
| Vernmark(22) | 2020 | Sweden | 54 | 379 | Rectum | Stage I–IV | 1.59 | 1.06–2.38 | 0.03 |  | 2.18 | 1.38–3.43 | <0.01 |
| Negri(47) | 2019 | Italy | 144 | 1214 | Colon and rectum | Stage I–IV | 1.03 | 0.79–1.34 | 0.81 |  |  |  |  |
| Tümay(48) | 2020 | Turkey | 48 | 324 | Colon and rectum | Stage I–IV | 1.02 | 0.63–1.66 | 0.94 |  |  |  |  |
| Arikan(49) | 2023 | Turkey | 16 | 41 | Left–sided colon and rectum | Stage IV | 2.04 | 1.02–4.08 | 0.04 |  |  |  |  |
| Huang(50) | 2022 | China | 58 | 472 | Colon and rectum | Stage I | 0.69 | 0.25–1.93 | 0.48 |  |  |  |  |
| Yan(7) | 2021 | China | 39 | 439 | Colon and rectum | Stage I–III | 0.82 | 0.50–1.33 | 0.42 |  |  |  |  |
| Lan(17) | 2021 | China | 73 | 219 | Colon and rectum | Stage I–IV | 1.91 | 1.281–2.859 | <0.01 |  |  |  |  |
| Kuan(51) | 2019 | China | 21 | 486 | Colon and rectum | Stage II | 2.76 | 1.41–5.40 | <0.01 |  | 3.23 | 1.43–7.29 | <0.01 |
| Li(52) | 2019 | China | 428 | 7502 | Colon and rectum | Stage I–III | 1.11 | 0.82‐1.51 | 0.46 |  |  |  |  |

MAC, mucinous adenocarcinoma. NMAC, non-mucinous adenocarcinoma. OS, overall survival. CSS, cancer-specific survival. DSS, disease-specific survival.
